# Transition Therapy: Tackling the Ecology of Tumor Phenotypic Plasticity

**DOI:** 10.1007/s11538-021-00970-9

**Published:** 2021-12-27

**Authors:** Guim Aguadé-Gorgorió, Stuart Kauffman, Ricard Solé

**Affiliations:** 1 ICREA-Complex Systems Lab, Universitat Pompeu Fabra, 08003 Barcelona, Spain; 2 Institut de Biologia Evolutiva, CSIC-UPF, 08003 Barcelona, Spain; 3 Institute for Systems Biology, Seattle, WA 98109 USA; 4 Santa Fe Institute, Santa Fe, NM 87501 USA

**Keywords:** Cancer ecology, Phenotypic switching, Epigenetic plasticity, Combination therapies, Transition therapy

## Abstract

Phenotypic switching in cancer cells has been found to be present across tumor types. Recent studies on Glioblastoma report a remarkably common architecture of four well-defined phenotypes coexisting within high levels of intra-tumor genetic heterogeneity. Similar dynamics have been shown to occur in breast cancer and melanoma and are likely to be found across cancer types. Given the adaptive potential of phenotypic switching (PHS) strategies, understanding how it drives tumor evolution and therapy resistance is a major priority. Here we present a mathematical framework uncovering the ecological dynamics behind PHS. The model is able to reproduce experimental results, and mathematical conditions for cancer progression reveal PHS-specific features of tumors with direct consequences on therapy resistance. In particular, our model reveals a threshold for the resistant-to-sensitive phenotype transition rate, below which any cytotoxic or switch-inhibition therapy is likely to fail. The model is able to capture therapeutic success thresholds for cancers where nonlinear growth dynamics or larger PHS architectures are in place, such as glioblastoma or melanoma. By doing so, the model presents a novel set of conditions for the success of combination therapies able to target replication and phenotypic transitions at once. Following our results, we discuss *transition therapy* as a novel scheme to target not only combined cytotoxicity but also the rates of phenotypic switching.

**Supplementary Information:**

The online version contains supplementary material available at 10.1007/s11538-021-00970-9.

## Introduction

Phenotypic plasticity is a widespread phenomenon across the tree of life. From bacteria to multicellular development, epigenetic pathways generate a population of diverse phenotypes from homogeneous, stable genomes (Sultan 2000; Piggliuci 2001; Margueron and Reinberg 2010; Balalszi et al. 2011). Phenotypic switching (PHS) is a stochastic phenomenon known to maintain population diversity in unicellular organisms as a means to survive in fluctuating environments (Kussell and Leibler 2005; Balaban et al. 2004). This mechanism can also be found to boost non-genetic heterogeneity in a special multicellular context: cancer cell populations (Flavahan et al. 2017). In this context, tumors can take advantage of already existing differentiation hierarchies to promote unlimited self-renewal or senescence and drug resistance with no need of selecting somatic mutations (Dean et al. 2005; Shackleton et al. 2009).

Phenotypic switching is a source for non-genetic heterogeneity in cancer beyond Cancer Stem Cells hierarchies (Flavahan et al. 2017; Marusyk et al. 2012; Brock et al. 2009). Beyond the well-known plasticity related to the Epithelial-Mesenchymal transition driving metastatic release (Kalluri and Weinberg 2009; Yeung and Yang 2017), more complex architectures with more than two switching phenotypes in place are being uncovered across cancers. A most recent example comes from Glioblastomas, where tumor cells are found to organize around four well-defined meta-modules resembling—though aberrant—healthy brain cell lines (Neftel 2019). This arrangement is highly robust: tumors initiated by single cells from a biopsy evolve toward the previous phenotypic composition, regardless of the specific phenotype of the original cell, showing that stochastic transitions happen between all of the four phenotypes. Similar dynamics have been described in breast cancer (Gupta et al. 2011), as well as in melanoma (Quintana 2008; O’connell and Weeraratna 2013) and prostate cancer (Jolly et al. 2018), and are nowadays considered key in the observation of non-Darwinian evolution of adaptive resistance across cancer types (Sharma 2010; Pisco et al. 2013; Su et al. 2017).

The existence of phenotypic plasticity in tumors has important consequences for therapy. Tumor relapse after therapy is usually acknowledged to be a consequence of pre-existing or acquired resistance mutations, present in a given subclone that survives and repopulates the tumor (see e.g., Diaz 2012). This image is often correct, yet further mechanisms in many therapeutic settings, from stem cell senescence (Jordan et al. 2006) to immunological editing (Sharma et al. 2017) prove that a wider scope is key when trying to understand therapeutic failure. The stochastic nature of switching between rogue cellular phenotypes allows robust and plastic tissue architectures, resulting in an adaptive mechanism that might be even harder to target (Sharma 2010). How does this affect therapeutic strategies? Models of phenotypic switching in cancer have helped in our understanding of metastatic dissemination (Gerlee and Nelander 2012; Jolly et al. 2017; Mathis et al. 2017), epigenetic drug combination (Alarcón et al. 2021) or the possible role of plasticity in maintaining one or more resistant phenotypes in place (Su et al. 2017; Jolly et al. 2017; Folguera-Blasco et al. 2019).

**Fig. 1:**
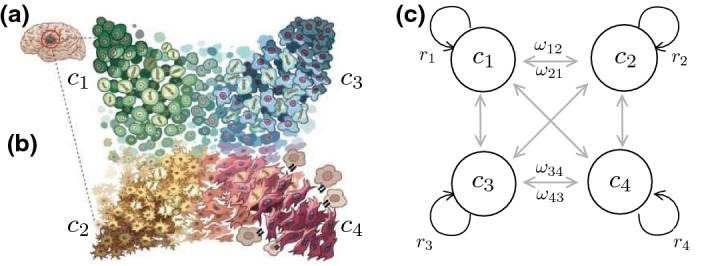
Phenotypic switching in cancer. Genetic analysis reveals four transitioning phenotypes in Glioblastoma (**a**) and thus a set of cancer cell populations (**b**, after Neftel 2019). Different transitions occur, linking phenotypes $C_k$ by means of a matrix of transition rates, as sketched in (**c**)

Here we present a toy model to study the characteristics of phenotypic plasticity in cancer by exploring the population dynamics of cellular replicators exhibiting transitions among them (Fig. 1). The model allows in particular to analyze the rise of the switching populations and the equilibrium conditions for stable heterogeneity in tumor scenarios involving uncontrolled cellular outgrowth, interphenotypic competition or nonlinear tissue-level growth constraints. Solving the ecological dynamics might render cues on the requirements to tumor extinction, with implications on novel therapeutic approaches when more than two phenotypes are in place.

## Phenotypic Switching Dynamics

In this section, we explore several features exhibited by different versions of a toy model of cancer cell populations exhibiting PHS. Our goal is to provide some basic bounds to the response of these systems to cytotoxic or targeted agents acting on the switching dynamics. Ecological models of heterogeneous cancer populations can be represented by means of a set of replicator equations (Nowak 2006). Consider a set of *N* phenotypes, where $\mathbf{{C}}=(C_1,...,C_N)$. The *i*-th cancer cell-type population will change in time following:

1 $$\begin{aligned} \frac{\mathrm {d}C_i}{\mathrm {d}t}= & {} \Gamma _i (\mathbf {C}) C_i \nonumber \\&+ \sum _{k \ne i} \omega _{ki} C_k-\sum _{k \ne i} \omega _{ik} C_i - C_i \phi (\mathbf {C}) \end{aligned}$$

with $(i,k=1,\ldots ,N)$. Here $\Gamma _i (\mathbf{C})$ indicates the functional form of the replication rate associated with the $i-$th clone, which in general will be a nonlinear function of clone or tumor size (Roose et al. 2007). The three last terms in the rhs correspond to (1) the phenotypic transitions from other phenotypes to phenotype $C_i$ (i.e., $C_k \rightarrow C_i$) (2) the complementary transitions from $C_i$ to the rest (i.e., $C_i \rightarrow C_k$) and (3) an outflow term that allows introducing competition and resource limitation effects. The previous set of equations can be re-written as follows:

2 $$\begin{aligned} \frac{\mathrm{d}C_i}{\mathrm{d}t}= \left( \Gamma _i (\mathbf{C}) -\sum _{k \ne i} \omega _{ik} \right) C_i + \sum _{k \ne i} \omega _{ki} C_k - C_i \phi (\mathbf{C}) \end{aligned}$$

By aggregating those terms affecting $C_i$, we can appreciate the fact that the effective growth rate of $C_i$ involves a trade-off between intrinsic replication and the likelihood that it shifts to a different cell type. However, a negative balance can be counterbalanced by the net inflow from the rest of the phenotypes holding $C_i$ in place. As a first approximation for rapidly growing cellular clones, a constant replication rate is associated to each phenotype (i.e., $\Gamma _i (\mathbf{C})= r_i$). We will later illustrate the effects of PHS under nonlinear growth dynamics by studying a particular example of tissue-level limitations in the Epithelial-Mesenchymal switch (Kalluri and Weinberg 2009; Yeung and Yang 2017).

What is the impact of PHS on potential therapeutic approximations? Are there novel attractors or alternative pathways to avoid targeted death? Relevant insight can be obtained by considering a first minimal system, where a finite set of cancer clones replicate at rate $r_i$, defined as the effective difference $r_i=b_i-d_i$ between birth $b_i$ and death $d_i$ rates, and that can be negative when cytotoxic therapy is effective (increasing death beyond birth, see Fig. 3a). In this section, we consider the simplest models of PHS in cancer populations.

**Fig. 2:**
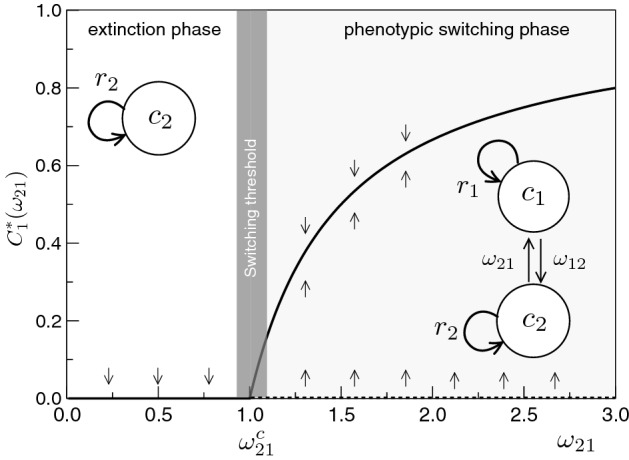
Bifurcation diagram for the reduced $N=2$ PHS model with two strains, as defined by Eq. (5) where the $C_1$ population is analyzed under CPC. This diagram represents the fixed points $C_1^*$ against the transition rate $\omega _{21}$. A critical switching threshold is defined here for a given $\omega _{21}^c$ separating a heterogeneous phase (gray) from a homogeneous one. Here $r_1=1,r_2=3/2$ and $\omega _{12}=1/2$, which gives a critical value $\omega _{21}=1.0$ (Eq. 8)

**Fig. 3:**
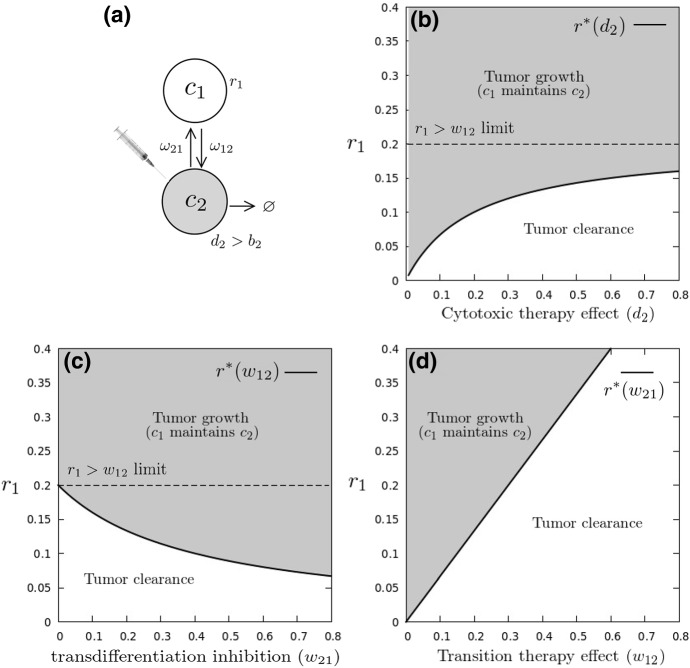
Transition therapy. Targeting proliferation of a single phenotype in a switching tumor (**a**). In the presence of PHS strategies, the resistant population $r_1$ is able to maintain tumor growth. Targeting sensitive cell death ($d_2$, **b**) or inhibiting transitions toward resistance ($w_{21}$, (**c**)) is likely to fail provided resistant cells replicate faster than they transition into the sensitive phenotype ($r_1>w_{12}$). PHS modeling indicates that only therapies draining $c_1$ into $c_2$ are effective across the whole parameter space (**d**)

### Predictable Heterogeneity in PHS Tumors

Experimental evidence in cancer populations exhibiting PHS shows that a secondary tumor evolves to the original phenotypic distribution of the primary malignancy, regardless of the initiating cell type (Neftel 2019; Gupta et al. 2011). This is an interesting outcome of PHS: the system has the potential to reliably restore population diversity in a predictable fashion. Instead of the often unpredictable heterogeneity driven by somatic mutations, we have here a surrogate of developmental dynamics driven by epigenetic changes. A first mathematical approach and its consequences are easily derived considering a population of two switchers ($N=2$) under a constant population constraint (CPC) (Balaban et al. 2004). Such CPC constraint allows for direct analysis of population fractions or densities $c_i = C_i/\sum _\mu C_\mu$ and writes

3 $$\begin{aligned} \frac{\mathrm{d}c_1}{\mathrm{d}t}= & {} (r_1 - w_{12})c_1 + w_{21}c_2 - c_1 \phi (\mathbf{C}) \end{aligned}$$

4 $$\begin{aligned} \frac{\mathrm{d}c_2}{\mathrm{d}t}= & {} (r_2 - w_{21})c_2 + w_{12}c_1 + - c_1 \phi (\mathbf{C}) \end{aligned}$$

This equation reduces to a simple competition model when $\omega _{ij}=0$. Darwinian selection would then be decided by the highest $r_i$, eliminating the possibility for heterogeneity.

Assuming constant population, the competition term reads $\phi (\mathbf{C}) = r_1 c_1 + r_2c_2$ and considering that $c_i$ are here densities and $c_1+c_2=1$, this is in fact the average replication rate, i.e., $\phi (\mathbf{C}) = \langle r \rangle$. Using this result, it is possible to reduce the system to a one-dimensional ordinary differential equation for the fraction of one of the populations, say $c_1$:

5 $$\begin{aligned} \frac{\mathrm{d}c_1}{\mathrm{d}t}= \gamma c_1 (1-c_1) - w_{12} c_1 \end{aligned}$$

with $\gamma = (r_1 - r_2 - w_{21})$. This model displays two fixed points, namely $c_1^*=0$ (extinction) and the heterogeneous point (where both populations persist) given by

6 $$\begin{aligned} c_1^*=1-{w_{12} \over \gamma } \end{aligned}$$

Interestingly, the presence of an heterogeneous attractor that is not dependent on initial phenotypic composition can be compared to experimental evidence of cell growth recapitulating original clonal distributions (Neftel 2019; Gupta et al. 2011). In particular, it can be seen that the attractor for population distributions, $c^*_1/c^*_2$, is consistent with the long-term stable distribution in the absence of intrinsic competition, $\text{ lim}_{t\rightarrow \infty } C_1(t)/C_2(t)$, because the CPC assumption is equivalent to formulating the model in terms of population concentrations (see SM). This result is consistent both analytically and through computer simulations, so that the minimal model is able to generate the basic *in vitro* properties of phenotypic switching. This, in turn, indicates that experimental observations of phenotypic distributions can be used to estimate the switching parameters that hold the heterogeneous cellular architecture, as previously seen in Gupta et al. (2011), Su et al. (2017), Goldman et al. (2015).

Under which conditions is the system able to maintain heterogeneity beyond the pressure of strictly-competitive Darwinian selection? The stability analysis of this system shows that heterogeneity will persist (i.e., $c_1^*,c_2^{*}>0$) and any initial condition will recapitulate the whole attractor distribution provided that

7 $$\begin{aligned} \omega _{21}- \omega _{12} > r_2-r_1. \end{aligned}$$

This inequality has an interesting, intuitive interpretation: $c_1$ will be positive, even if $r_2>r_1$, provided that the difference between transition rates is larger than the difference between growth rates, highlighting the ability of PHS to maintain tumor heterogeneity (Fig. 2). This allows defining a threshold value: heterogeneity will be observed when

8 $$\begin{aligned} \omega _{21}^c = \omega _{12}+(r_2-r_1) \end{aligned}$$

which determines the threshold condition for the switching rate $\omega _{21}$ required to sustain $C_1$, being other parameters fixed. The basic bifurcation diagram associated to this model is shown in Fig. 2. Two phases are indicated. The first is associated to the diverse switching phenotypes (for $\omega _{21}>\omega _{21}^c$, gray area). Here a single attractor exists, which can be reached from any initial condition. Another, homogeneous phase occurs for $\omega _{21}<\omega _{21}^c$ where only the fastest replicating population persists.

The transition defines a tipping point that is determined (with other parameters fixed) by the rate of recovery provided by the PHS mechanism. The diagram is obtained under unfavorable replication: we use $r_1<r_2$ which, in the absence of PHS, would inevitably lead to the extinction of $C_1$. The presence of a heterogeneous phase indicates that phenotypic populations can persist even in unfavorable competition scenarios. How does the system evolve when these populations are targeted by therapy?

### PHS in the Sensitive-Resistant Scenario

A first instance of PHS in cancer is observed in tumors deploying temporary resistant cell subpopulations (Sharma 2010). In certain settings, such drug-tolerant phenotypes can arise in the absence of resistance-conferring alterations (Talpaz et al. 2002; Berrieman et al. 2004), indicating the role of non-Darwinian epigenetic plasticity in generating and maintaining tolerant phenotypes in place (Goldman et al. 2015). Modeling PHS can uncover the underlying dynamics of sensitive-resistant populations, proposing specific therapeutic outlines.

In order to formulate this model, we remove the competition term $c_i \phi (\mathbf{C})$ in the previous Eqs. (3–4) and consider phenotypic populations away from their carrying capacity. Now $C_i$ are not densities, but actual population counts. We study the following linear system

9 $$\begin{aligned}&\frac{\mathrm{d}C_1}{\mathrm{d}t}= (r_1 - w_{12})C_1 + w_{21}C_2 \end{aligned}$$

10 $$\begin{aligned}&\frac{\mathrm{d}C_2}{\mathrm{d}t} = w_{12}C_1 + (r_2 - w_{21})C_2 \end{aligned}$$

The unbounded system does not admit a single-population solution: the tumor either gets extinct or both $C_1(t)$ and $C_2(t)$ undergo exponential growth. As previously discussed, long-term phenotypic composition $C_1/C_2$ is still predictable and independent from initial conditions (see SM), as observed in experimental setups (Neftel 2019; Gupta et al. 2011). We know that the (0, 0) attractor is stable if both *effective* growth rates are negative. Since $r_i = b_i - d_i$, this can be true if death rates for both cell types are increased beyond their birth rates by means of two different drugs. However, provided $C_1$ is a drug-tolerant state (Sharma 2010), chemotherapy will only increase death rates of the $C_2$ population.

Let us introduce a nomenclature for cytotoxic-sensitive and -resistant phenotypes. Assume that cell type $C_1$ has a positive replication rate $r_1>0$ under chemotherapy. In this setting, the drug-resistant phenotype will be labeled $C_\mathrm{R}$, growing at rate $r_\mathrm{R}$. The death rate of cell type $C_2$ can be increased by means of a cytotoxic therapy, so that $r_2 = b_2 - d_2$ could shift from positive to negative (Fig. 3a), and be labeled $C_\mathrm{S}$, with $r_\mathrm{S}=b_\mathrm{S} - d_\mathrm{S}<0$. The sensitive-resistant system now writes

11 $$\begin{aligned}&\frac{\mathrm{d}C_R}{\mathrm{d}t}= (r_\mathrm{R} - w_{\mathrm{RS}})C_\mathrm{R} + w_{\mathrm{SR}}C_\mathrm{S} \end{aligned}$$

12 $$\begin{aligned}&\frac{\mathrm{d}C_S}{\mathrm{d}t} = (r_\mathrm{S} - w_{\mathrm{SR}})C_\mathrm{S} + w_{\mathrm{RS}}C_\mathrm{R} \end{aligned}$$

Stability analysis of the tumor-free attractor results in a threshold replication rate for $C_\mathrm{R}$ (see SM),

13 $$\begin{aligned} r_\mathrm{R}^{*} = {\displaystyle \frac{\displaystyle w_{\mathrm{RS}}}{\displaystyle 1+\left( {w_{\mathrm{SR}} \over \left| r_\mathrm{S}\right| } \right) }} \end{aligned}$$

If $C_\mathrm{R}$ replicates faster than this threshold level, it will repopulate the tumor and maintain the sensitive population $C_\mathrm{S}$ (Fig. 3). This is consistent with recent analytical results from (Gunnarsson et al. 2020) for the progression of a tumor in the presence of a drug-tolerant phenotype.

This result uncovers several potential therapeutic implications. In the setting that $C_\mathrm{R}$ is a drug-tolerant phenotype, therapy could focus on increasing $d_S$, the death rate of the sensitive phenotype (Tracqui et al. 1995), decreasing $w_{\mathrm{SR}}$, the rate at which the sensitive phenotype becomes resistant (Hari et al. 2020), or increasing $w_{\mathrm{RS}}$, the rate at which the resistant phenotype transdifferentiates into drug-sensitivity (Goldman et al. 2015). All approaches could potentially drive tumor extinction (Fig. 3).

However, if the drug-tolerant phenotype replicates faster than its transition rate ($r_\mathrm{R}>w_{\mathrm{RS}}$), which is a plausible setting considering measured $w_{ij}$ rates in some cellular substates (Gupta et al. 2011), any efforts on $d_\mathrm{S}$ or $w_{\mathrm{SR}}$ will fail at eliminating the tumor (Fig. 3b, 3c). Mathematically, Eq. (11) implies a minimal resistant-sensitive transition rate, below which the resistant population persists:

14 $$\begin{aligned} w_{\mathrm{RS}}^* = r_\mathrm{R} \left( 1+\theta _\mathrm{S}\right) \end{aligned}$$

with $\theta _S = w_{\mathrm{SR}}/|r_\mathrm{S}|$ being the transition-to-death ratio of the sensitive population. In very effective therapy settings, $\theta \sim 0$ and $w_{\mathrm{RS}}^* = r_\mathrm{R}$. The only path to eliminating the drug-resistant tumor is by increasing its transition rate beyond the threshold cycling rate.

This threshold has potential implications on switching inhibition, in that therapies targeting inhibition of sensitive-resistant transitions ($w_{\mathrm{SR}}\sim 0$) are likely to fail unless the same drug alters $r_\mathrm{R}$ or $w_{\mathrm{RS}}$. This is a key result regarding therapeutic options targeting EMT inhibition to prevent metastases (Tripathi et al. 2020; Ramesh et al. 2020).

Another particular example here is provided by the discovery of sensitive transient states in chemotherapy experiments on breast cancer (Goldman et al. 2015). In them, resistance to first-line chemotherapy implies a transition to a transient phenotype *T* that can be resensitized by a second drug. Initial chemotherapy increases $w_{\mathrm{RT}}$, while the second drug resensitizes this transient state to initial chemotherapy, inducing $w_{\mathrm{TS}}$. The overall effect is that of a combination scheme that increases $w_{\mathrm{RS}}$. In the specific setting of Goldman et al., the measured transition rates from stem-cells to the induced state is $w_{\mathrm{RT}}\approx 0.96$day$^{-1}$, while $r_{\mathrm{R}}\approx 0.5$day$^{-1}$, so that therapeutic efficacy correlates with the *transition threshold* condition (14). To which extent is this specific therapeutic approach robust across cancer types?

Our results highlight the potential limitation to be accounted for when designing such PHS therapeutic strategies: increasing $w_{\mathrm{RS}}$, the rate at which $C_\mathrm{R}$ switches to $C_\mathrm{S}$, can drain the replicative phenotype into the one we can kill by cytotoxic therapy (Fig. 3d), only if it overcomes $C_\mathrm{R}$ replication. Transition to a sensitive state will only be effective if the resistant state cannot persist and maintain the PHS architecture.

A therapeutic corollary of this is that a most effective combination therapy in a sensitive-tolerant setting would contemplate increasing $w_{\mathrm{RS}}$ while also decreasing $r_\mathrm{R}$ to facilitate the threshold condition. Even if initial cytotoxic efforts might not slow down $C_\mathrm{R}$ replication, other specific microenvironmental cues, in the form of antiangiogenic (Ledzewicz and Schattler 2007) or dormancy-inducing (Goss and Chambers 2010) drugs targeting cell cycling rate are likely to help the overall *transition therapy* scheme.

### PHS in Nonlinear Growth Scenarios: Epithelial-Mesenchymal Plasticity

We have studied so far the role of PHS in allowing the growth of cellular phenotypes under linear replication motifs (Eqs. 3–4, 11–12). This simplification on tumor growth dynamics allows the uncovering of certain key thresholds, related with the ability of PHS as a whole-tumor strategy to overcome the pressures of competition (Eq. 8) or cytotoxic therapy (Eqs. 13–14).

However, cancer populations are known to follow markedly nonlinear growth dynamics (Benzekry et al. 2014). How do PHS strategies modulate the growth and survival of tumors in the presence of nutrient and spatial constraints hampering exponential replication?

We here propose to explore PHS in nonlinear growth scenarios by studying a minimal model of the Epithelial-Mesenchymal plasticity (EMP), a fundamental example of non-genetic heterogeneity in cancer (Kalluri and Weinberg 2009). On a first approach, EMP involves two reversible PHS processes^1^: the Epithelial-Mesenchymal transition (EMT), in which Epithelial cells lose their polarity and cell–cell adhesion, hereby gaining enhanced migratory capacity and invasiveness leading to metastatic dissemination (Yeung and Yang 2017), and the Mesenchymal-Epithelial transition (MET) involving the opposite process.

**Fig. 4:**
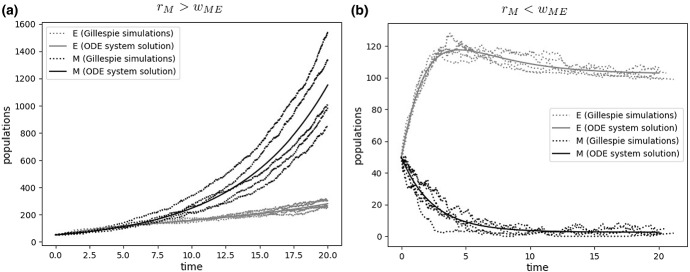
Transition therapy in populations under nonlinear growth. Ecological models of phenotypic switching highlight an important scenario: An exponentially growing phenotype (here, a mesenchymal phenotype *M* growing beyond tissue boundaries) can be indirectly controlled by the carrying capacity of a limited phenotype (here, an epithelial phenotype *E* constrained by spatial and nutrient limitations) following a brief phase where *E* exceeds its carrying capacity due to temporary increased seeding by *M*. Separation between cell population outgrowth (**a**) and tumor control (**b**) is given by a sharp threshold in the growth-to-switching ratio of the exponential phenotype

To elucidate the ecological dynamics of this reversible process we propose a simplified dynamical setting, where the Epithelial phenotype grows following logistic dynamics, indicative of spatial constraints at the tissue level (Gatenby 1991). The Mesenchymal phenotype, through the loss of cell–cell adhesion (Yeung and Yang 2017), can be approximated, during a first phase of rapid *metastatic release* (Schop et al. 2008), to become released from carrying capacity limitations and grow exponentially.

15 $$\begin{aligned} \frac{\mathrm{d}E}{\mathrm{d}t}= & {} \left( r_\mathrm{E}\left( 1-\beta _E E\right) - w_{\mathrm{EM}} \right) E + w_{\mathrm{ME}}M \end{aligned}$$

16 $$\begin{aligned} \frac{\mathrm{d}M}{\mathrm{d}t}= & {} w_{\mathrm{EM}}E + (r_\mathrm{M} - w_{\mathrm{ME}})M \end{aligned}$$

Here, $\beta$ is the inverse of the carrying capacity of the epithelial tissue, so that the Mesenchymal phenotype is considered to grow at $\beta \approx 0$ during the modeled phase (Schop et al. 2008). PHS is introduced as a stochastic switch at average rates $w_{\mathrm{EM}}$ and $w_{\mathrm{ME}}$, respectively.

What are the potential ecological outcomes of this scenario, and how do they differ from the previous models? A common treatment approach focuses on blocking the EMT, by reducing $w_{\mathrm{EM}}$ in the aim of minimizing metastatic dissemination (Ramesh et al. 2020). Is this the most effective approach?

Several key results follow from studying the attractor states. Beyond $(E^{*},M^{*})=(0,0)$, a novel coexistence attractor not seen in the model (11–12) appears:

17 $$\begin{aligned} E*= & {} \frac{1}{\beta }\left[ 1-\frac{w_{\mathrm{EM}}}{r_\mathrm{E}}\left( 1 -\frac{w_{\mathrm{ME}}}{w_{\mathrm{ME}}-r_{\mathrm{M}}} \right) \right] \end{aligned}$$

18 $$\begin{aligned} M^{*}= & {} \frac{w_{\mathrm{EM}}}{w_{\mathrm{ME}}-r_{\mathrm{M}}} E^{*} \end{aligned}$$

The attractor state at the Epithelial level indicates a stable population, that could exceed the carrying capacity of the tissue provided that $w_{\mathrm{ME}}>r_\mathrm{M}$ or else become increasingly small until $E^{*}=0$. A more interesting scenario appears from looking at $M^{*}$. In the absence of PHS or at least $r_\mathrm{M}>w_{\mathrm{ME}}$, the mesenchymal population will grow exponentially (or decay for $r_\mathrm{M}<0$), potentially initiating metastatic disease (Fig. 4a). However, PHS allows for a novel attractor state, where both populations are controlled, even for $r_\mathrm{M}>0$, if $w_{\mathrm{ME}}>r_\mathrm{M}$ (Eq. 18, Fig. 4b).

This novel scenario, characterized by the presence of nonlinear growth dynamics resulting from tissue-level constraints (Gatenby 1991), indicates that mesenchymal cells, even if replicating under no growth constraints, could be indirectly controlled by the growth limitation of the epithelial population, provided they are drained by a combined cytotoxic+transition treatment achieving $w_{\mathrm{ME}}>r_ {\mathrm{M}}$ (Fig. 4). This alternative version of *transition therapy* highlights how targeting the rate of the MET through microRNAs (Yao et al. 2011; Wang et al. 2014) (instead of reducing the rates of the EMT switch (Ramesh et al. 2020)) can provide a novel opportunity toward controlling initial metastatic release in the presence of EMP.

### Targeting PHS in Larger Architectures ($N>2$)

We have used the $N=2$ case to illustrate the concept of cancer growth with switching and how different growth-transition trade-offs can influence therapeutic outcome in simple Sensitive-Resistant scenarios. But tumor architectures often include more than two coexisting phenotypes (Neftel 2019; Gupta et al. 2011) beyond the effects of chemotherapy. Given a larger system with *N* phenotypes that switch stochastically, can our mathematical framework define the limits of PHS resilience? The analytical approach for $N>2$ independent phenotypes becomes harder as we add dimensions, and results now depend on $N^{2}$ parameters. However, certain average effects of given therapy schemes can be predicted under symmetry assumptions.

Let us here suppose a common therapeutic scheme, where certain phenotypes are sensitive to a first drug, while others tolerate it. This common scenario can be encountered in the development of adaptive resistance to docetaxel (DTX) in breast cancer (N=3, (Gupta et al. 2011; Goldman et al. 2015)) or the targeting of either EGFR, PDGFRA, or CDK4 only affecting one out of four phenotypes in Glioblastoma (N=4, (Neftel 2019)).

The problem can be tackled as follows. Let us first consider the $N=3$ case, as indicated in Fig. 3a. In order to reduce the complexity of our calculations, we consider a coarse-graining assumption: all resistant and sensitive cells do so at equal rates, $r_\mathrm{R}$ and $r_\mathrm{S}$, respectively, and transition rates between replicating and dying cells are also homogeneous. This assumption is summarized in Fig. 3b.

In this scenario, suppose a system with two phenotypes that replicate at $r_\mathrm{R}>0$ and hold a sensitive phenotype $r_\mathrm{S} <0$:

19 $$\begin{aligned}&{\text {d}C_1\over \text {d}t}= ( r_\mathrm{R} - w_\mathrm{RR} -w_\mathrm{RS}) C_1 + w_\mathrm{RR}C_2 + w_\mathrm{SR} C_3 \end{aligned}$$

20 $$\begin{aligned}&{\text {d}C_2\over \text {d}t}= ( r_\mathrm{R} - w_\mathrm{RR} -w_\mathrm{RS}) C_2 + w_\mathrm{RR}C_1 + w_\mathrm{SR}C_3\end{aligned}$$

21 $$\begin{aligned}&{\text {d}C_3\over \text {d}t}= (r_\mathrm{S} - 2 w_\mathrm{SR})C_3 + w_\mathrm{RS} (C_1+C_2) \end{aligned}$$

**Fig. 5:**
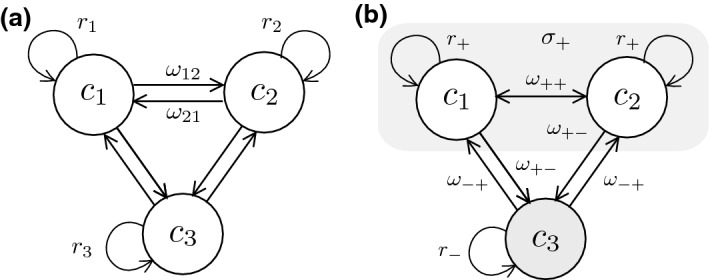
Transitions for N=3 phenotypes. For a $N=3$ case study, the flow diagram (**a**) indicates all the transition and replication rates. In order to determine the requirements for successful therapy when a cytotoxic drug is used against $C_3$, a homogeneous model (**b**) is used. $+$ subscripts refer to therapy tolerant or resistant phenotypes $C_R$, while − indicate those phenotypes with negative effective replication under the action of a drug

Let us now indicate by $\sigma _R$ the total population of resistant cells, i.e., $\sigma _\mathrm{R}=C_1+C_2$ (Fig. 3b). In this case, the system reduces to

22 $$\begin{aligned}&{\mathrm{d}\sigma _\mathrm{R} \over \mathrm{d}t}= \sigma _\mathrm{R} r_\mathrm{R} - \sigma _\mathrm{R} w_{\mathrm{RS}} + 2 w_{\mathrm{SR}} C_3 \end{aligned}$$

23 $$\begin{aligned}&{\mathrm{d}C_3\over \mathrm{d}t}= C_3 r_\mathrm{R} - 2C_3 w_{\mathrm{SR}} + w_{\mathrm{RS}} \sigma _\mathrm{R} \end{aligned}$$

For this two-compartment system, it can be shown that the minimal threshold for the resistant population replication rate is:

24 $$\begin{aligned} \displaystyle {r_\mathrm{R}^{*} = \frac{w_{\mathrm{RS}}}{ \left( 1 + 2{\displaystyle \frac{\displaystyle w_{\mathrm{SR}}}{\displaystyle |r_\mathrm{S}|}}\right) } }. \end{aligned}$$

This calculation, under our homogeneity assumptions, can be done in a systematic way for a switching population with of *N* cell types (see SM). Specifically, we can consider $n_\mathrm{R}$ replicators with a positive effective growth rate $r_\mathrm{R}$ and $n_\mathrm{S}$ sensitive cell types targeted by therapy, so that their death rate increases beyond birth and $b_\mathrm{S} - d_\mathrm{S} = r_\mathrm{S}<0$.

By aggregating the two different populations in $\sigma _\mathrm{R}$ and $\sigma _\mathrm{S}$ compartments, the problem of a tumor with *N* switching phenotypes can be studied (see SM). It can be shown that the minimal growth rate for the positive replicators to sustain the tumor is

25 $$\begin{aligned} \displaystyle {r_\mathrm{R}^{*}(w_{\mathrm{RS}}, n_\mathrm{S}) = n_S {\displaystyle \frac{\displaystyle w_{\mathrm{RS}}}{\displaystyle \left( 1 + (N-n_S)\frac{ w_{\mathrm{SR}}}{|r_\mathrm{S}| }\right) }} }. \end{aligned}$$

Complete cancer eradication can happen if all phenotypes are targeted. Targeting less than four phenotypes can prove useless if the other cell types maintain diversity by replicating faster than (3) (Fig. 5). Through sequentially targeting several phenotypes, we can increase $n_S$ and decrease $n_\mathrm{R}=N-N_\mathrm{S}$ accordingly. This therapeutic intervention results in a nonlinear increase in the pressure to maintain diversity and growth (Fig. 5a).

The existence of a threshold relating replication (*i.e.,* drug sensitivity), targeted phenotypes and phenotypic transitions to overall therapy effectivity is consistent with results in Goldman et al. (2015), where several combinations of drugs inhibiting plasticity-mediated resistance are tested in BRAF mutant melanoma. There is direct correlation between the effect of drugs on transition rates and overall cellular growth, with failure of vemurafenib-only therapy related with $r_\mathrm{R}$ overcoming the threshold (21) for all plastic populations (Goldman et al. 2015).

**Fig. 6:**
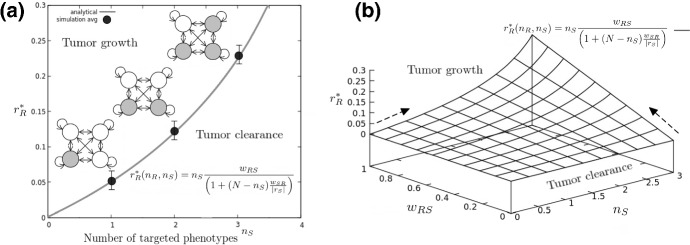
PHS therapy in larger architectures ($N>2$). In **a**, replicating phenotypes (empty circles) maintain drug-targeted phenotypes (gray) through stochastic switching. Therapies targeting replication through targeting of genetic drivers such as EGFR in GBM are likely to increase nonlinearly the cost for resistance phenotypes to maintain diversity (continuous line, equation displayed in the inset). Stochastic Gillespie simulations result in a certain degree of deviation, where extinction in smaller populations can eventually happen for values of $r>r_\mathrm{R}$. Filled dots indicate the value for which 95% of the computational experiments result in population extinction, with error bars indicating 5% deviations from this value (see SM for computational details). In **b**, the effect of combined transition therapies draining *R* phenotypes into *S* is captured by a *therapeutic efficacy* landscape. In it, the effect of adding a transition therapy ($w_{\mathrm{RS}}$) or a novel targeted agent ($n_\mathrm{S}$) is captured by the gradients (dark arrows), directly resulting in an analytical scheme to compute best scenarios for combination therapy in PHS (Eqs. 27,28)

For a GBM setting, the threshold could be potentially exploited by a multi-gene, multi-drug approach able to target the three main genetic pathways of the AC-like, OPC-like and NPC-like populations through EGFR, PDGFRA and CDK4, respectively (Neftel 2019). Each novel target is likely to induce a strong pressure for replication to resistant phenotypes $\sigma _\mathrm{R}$, eventually resulting in the mesenchymal phenotype alone bearing the pressure of the replication threshold ($n_\mathrm{S}=3$, Fig. 6a). This is a specially relevant result, since it provides a rough estimate of the potential obstacles to replication-oriented therapy posed by the presence of *N*-dimensional switching.

What is the role of transition rates in therapeutic schemes for $N>2$? We know from the smaller system (11–12) that increasing $C_\mathrm{R}$ draining beyond $w_{\mathrm{RS}}\ge r_{\mathrm{R}}$ is a necessary condition for tumor eradication. When *N* phenotypes are at place, the condition for *Transition therapy* to success writes:

26 $$\begin{aligned} w_{\mathrm{RS}} \ge \frac{r_\mathrm{R}}{n_\mathrm{S}}\left[ 1+(N-n_\mathrm{S})\frac{ w_{\mathrm{SR}}}{|r_\mathrm{S}|} \right] \end{aligned}$$

The multiple phenotypes architecture threshold differs from Eq. (14) when $n_\mathrm{S}$ is considered. This result implies a novel combination therapy landscape, able to characterize overall therapeutic effectivity as a function of the parameter changes occurring after each drug hit (Fig. 6b). The landscape offers the possibility of computing the gradients (Fig. 6b), dark arrows) that indicate the pressure on $r^{*}_\mathrm{R}$ exerted by either increasing $w_{\mathrm{RS}}$ or $n_\mathrm{S}$. In given therapy settings, computing this landscape and its gradients result in a preliminary indicator on choosing if the next drug should focus on draining the untargeted phenotype ($w_{\mathrm{RS}}$) or targeting a novel sensitive phenotype ($n_\mathrm{S}$). Overall, this could improve targeting of multi-phenotype plastic networks where $w_{ij}$ is only targeted so far through inhibition and not increase (Goldman et al. 2015).

The gradients of $\partial _{w_{\mathrm{RS}}} r^*$ and $\partial _{n_{S}} r^*$ therefore indicate a key evolutionary ingredient for combination therapeutic designs

27 $$\begin{aligned} \frac{\partial r^{*}_\mathrm{R}}{\partial w_{\mathrm{RS}}} = {\displaystyle \frac{\displaystyle n_\mathrm{S}}{\displaystyle \left( 1 + (N-n_\mathrm{S})\frac{ w_{\mathrm{SR}}}{|r_\mathrm{S}| }\right) }} , \end{aligned}$$

28 $$\begin{aligned} \frac{\partial r^{*}_\mathrm{R}}{\partial n_{\mathrm{S}}} = {\displaystyle \frac{\displaystyle w_{\mathrm{RS}}\left( 1+N\frac{ w_{\mathrm{SR}}}{|r_\mathrm{S}| } \right) }{\displaystyle \left( 1 + (N-n_\mathrm{S})\frac{ w_{\mathrm{SR}}}{|r_\mathrm{S}| }\right) ^2}} . \end{aligned}$$

With given parameters (Table 1), adding single agents should follow from which gradient of both is larger. If not, using drugs that induce small gradient effects on $r_\mathrm{R}^*$ is likely to allow resistant phenotypes a window to explore escape mechanisms in the lack of strong drug activity (Liau et al. 2017).

## A Note on PHS and Evolutionary Game Theory

Evolutionary Game Theory (EGT) provides an optimal framework to understand the evolution of species or phenotypes in a population, based on the notion that their *fitness* (usually, replication rate) depends on the interaction with other species and their abundances in a given ecological setting (Smith and Price 1973; Weibull 1997). Among many other applications (see e.g., (Weibull 1997) for an extensive review), EGT provides a framework to understand the relative abundances of species, here seen as *strategies* played in the ecological game. Knowing this, one may ask if EGT is a useful tool to model the phenotypic dynamics observed in tumors with evidence of PHS.

**Table 1:** Parameter description and approximated experimental values for the ecology of phenotypic switching. The replication of cancer cells and the fitting of tumor growth curves with nonlinear models has been studied for decades (see e.g., (Benzekry et al. 2014) and references therein). More recently, evidence for spontaneous and stochastic phenotypic transitions in cancer cells (Neftel 2019; Gupta et al. 2011; Su et al. 2017; Goldman et al. 2015) indicates that transition rates might happen at a rate inferior to that of malignant cell replication

Parameter	Description	Experimental values	References
$r_i$	Phenotype *i* replication rate	0.2 $\sim$ 0.8 days$^{-1}$	Benzekry et al. (2014) and refs. therein
$w_{ij}$	Rate of phenotypic transition $i\rightarrow j$	(0.01 $\sim$ 0.5)$\times r_i$ days$^{-1}$	(Gupta et al. 2011; Goldman et al. 2015)
*K*	Carrying capacity of the cancerous tissue	$2\times 10^9$ $\sim$ $4\times 10^{10}$	(Kuznetsov et al. 1994; Wilkie and Hahnfeldt 2013)

Very broadly, EGT can be brought up through the notion of the so-called Replicator-Mutator model, where the abundance dynamics of phenotype $x_i$ follow

29 $$\begin{aligned} \frac{\mathrm{d}x_i}{\mathrm{d}t} = \sum _{j=1}^{N} x_j f_j (\mathbf {x}) Q_{ji} - x_i\phi (\mathbf {x}), \end{aligned}$$

where $f_j(\mathbf {x})$ stands for the replication rate of phenotype *j* given the abundances of all phenotypes in the population (often expressed as a Payoff Matrix), $Q_ji$ is the mutation rate from phenotype *j* to *i*, and the last term accounts for the competition dynamics associated to the ecosystem. By applying this dynamical framework, one could predict the final distribution of phenotypes in an ecosystem by knowing how each of these phenotypes (often representing species) interacts when finding any other (Weibull 1997).

EGT has been successfully applied to several open problems in mathematical oncology (Pacheco et al. 2014), where it has been used to target ecological phenomena such as intra-tumor heterogeneity and tumor cell metabolism (Basanta and Anderson 2013) or the effect of anti-cancer treatments (Swierniak et al. 2019). We hypothesize that EGT will also be a candidate framework to study the dynamics and implications of PHS beyond what has been studied in this work. To do so, however, two main issues will need to be solved.

On the one hand, Replicator-Mutator dynamics are based on the notion that changes between strategies (phenotypic mutations) happen during replication (as expressed in $f_j (\mathbf {x}) Q_{ji}$), as errors in cellular DNA occur during cell division (Negrini et al. 2010). The stochastic nature of phenotypic transitions and epigenetic switching, however, spans a much broader scenario, where transitions can happen at any time, hereby changing the overall dynamics into those of Eqs. 1–2.

More importantly, solving EGT models requires knowledge on the exact payoff matrix: the benefit or cost that a given phenotype obtains when encountering another (Pacheco et al. 2014; Basanta and Anderson 2013). Evidence for phenotypic switching architectures with more than two phenotypes in cancer is still very recent (Neftel 2019; Gupta et al. 2011; Su et al. 2017; Goldman et al. 2015). In this context, further experimental insight rendering a clear picture of the ecological cues of phenotypic interactions could bring up a first, preliminar description of *PHS payoff matrices*, that combined with stochastic switching dynamics make up for a full model (Eqs. 1–2) that would represent a breakthrough in our capacity to understand, and hence modulate with therapy, epigenetic switching in cancer.

## Discussion

Several considerations on therapy design arise directly from the previous results (and our simplifying assumptions). A well-adapted population can maintain non-adapted cell types, provided replication and transition rates are tuned accordingly. Evidence for skewness in experimental transition rate values (Gupta et al. 2011; Su et al. 2017) could indicate the evolution of PHS networks toward enhancing well-adapted phenotypes. PHS offers therefore an alternative pathway to cancer heterogeneity and consequent drug resistance (Easwaran et al. 2014). In this context, single-phenotype strategies are likely to fail, steering tumor evolution toward other phenotypes instead of providing a cure. Our mathematical framework provides a qualitative understanding of such failure for *N*-dimensional PHS architectures.

In them, what is to be tackled is diversity itself: if only one phenotype can be targeted, the model indicates that others can be drained by increasing the rates at which they transition to the dying one. A key implication here is that inhibition of resistant-phenotype transitions is not necessarily a successful approach if drug-tolerant cell types are not specifically drained toward sensitivity.Furthermore, PHS provides a therapeutic opportunity when different nonlinear growth dynamics are in place. As a case example, we have explored the possibility of controlling an exponentially-growing mesenchymal phenotype by increasing phenotypic switching toward a sibling Epithelial population, whose growth is limited by tissue-level spatial and nutrient limitations.

Therapeutic strategies that target differentiation pathways are already in place (de Thé 2018), and much is known about dedifferentiation and reprogramming across cell types (Jopling et al. 2011; Huang 2009). Clinical and experimental evidence points to differentiation-regulating genes as potential targets of *transition therapy*. Potential examples are TBX3 affecting inter-phenotype switching (Gupta et al. 2011) or SFK/Hck regulating chemotolerance (Goldman et al. 2015) in breast cancer cell lines. Epigenetic drugs targeting DNA methylation are nowadays another therapeutic opportunity (Gore et al. 2006; Juergens et al. 2011), and combinatorial antibody libraries as regulators of cell fate (Lerner et al. 2015) or stem cell transdifferentiation (Xie et al. 2013) might provide further options to induce phenotypic transdifferentiation as a therapeutic strategy. Recent evidence indicates the relevance of obtaining a clear portrait of the underlying Gene Regulatory Networks (GRNs) driving plasticity in order to target possible feedback loops or hysteresis mechanisms of PHS (Hari et al. 2020; Celià-Terrassa et al. 2018). Furthermore, the possibility that phenotypic switching can be targeted beyond oncogenic phenotypes (Ishay-Ronen et al. 2019) opens up the Waddington landscape (Waddington 1957) to be explored by *transition therapy*.

When more than two phenotypes coexist it is likely that several cell types have evolved oncogenic advantage (Neftel 2019; Su et al. 2017). Our approach indicates that targeting several phenotypes increases nonlinearly the pressure for tumor survival. Drug combination targeting multiple cell types together with transition rates to drain non-targeted phenotypes could result in increased benefits for patient survival if specific PHS threshold conditions are fulfilled.

Sequential therapy schemes are known to drive tumor evolution by inducing pressures that drive clonal selection (Gatenby and Brown 2018). Even in tumors where phenotypes show self-renewal capacity after cytotoxic therapy, our modeling approach is a predictive tool for the resulting phenotypic trajectories. Since we can compute the stable phenotypic composition for any combination of parameters, knowing how they change after therapy results in a quantitative prediction of the new tumor state.

Our framework, therefore, can prove helpful to understand tumor evolution after each drug (Goldman et al. 2015; Amirouchene-Angelozzi et al. 2017). Sequential drug effects have been studied for tumors under clonal evolution schemes (Bozic 2013), but accumulated knowledge indicates that epigenetic plasticity introduces novel conditions for eradication of resistant cell types (Easwaran et al. 2014). The ability to push the system toward equilibria predicted by our model puts forward the opportunity of directing evolution to pre-sensitize the tumor to a second drug (Basanta et al. 2012). Following the notion of cancer attractors and combination therapy (Huang et al. 2009; Huang and Kauffman 2013), increasing (instead of only inhibiting) transition rates offers a new way of thinking in how to tackle PHS-driven heterogeneity under a more “developmental” perspective. Future extensions might need to be considered, such as putting together our ecological perspective with that of single-cell developmental reprogramming (Alarcón et al. 2021; Folguera-Blasco et al. 2019) or including the dynamical effects of spatially explicit structure (Strobl et al. 2021), niche architecture or tissue hierarchy (Solé and Aguadé-Gorgorió 2021). Each extra layer will undoubtedly modify our basic bounds, but we conjecture that the ways PHS influences tumor responses will be basically the same.

## Supplementary Information

Below is the link to the electronic supplementary material.

Supplementary material 1 (pdf 1895 KB)

## Data Availability

Data sharing not applicable to this article as no datasets were generated or analyzed during the current study.

## Footnotes

1 A third intermediate phenotype, termed *hybrid Epithelial/Mesenchymal*, has been observed to play a key role in metastatic dissemination, with hybrid E/M cells characterized by invasive traits but also a certain degree of collective migratory behavior allowing the formation of Circulating Tumor Cell clusters (Jolly et al. 2018)
