# Supplementary material for: Transition Therapy: Tackling the Ecology of Tumor Phenotypic Plasticity
Source: Bull Math Biol. 2021 Dec 27;84(1):24. doi: 10.1007/s11538-021-00970-9 (PMC8712307; doi:10.1007/s11538-021-00970-9)
Supplement: Supplementary file 1 — Supplementary material 1 (pdf 1895 KB) [file 11538_2021_970_MOESM1_ESM.pdf]

Supplementary Matherial to:
Transition therapy: tackling the ecology of tumor phenotypic plasticity

Guim Aguadé-Gorgorió,[1,2] Stuart Kauffman,[3] and Ricard Solé*[1,2,4]
[1]ICREA-Complex Systems Lab, Universitat Pompeu Fabra, 08003 Barcelona, Spain
[2]Institut de Biologia Evolutiva, CSIC-UPF, 08003 Barcelona, Spain
[3]Institute for Systems Biology, Seattle WA 98109, USA
[4]Santa Fe Institute, Santa Fe NM 87501, USA

A. Any starting cell can recapitulate the original phenotypic composition

In this section we aim at proving how any initial phenotypic configuration recapitulates the same final distribution. Following the main text, this can be both computed through a constant population constraint (CPC) model or by computing the long therm population equilibrium $\lim_t \to \infty C_1(t)/C_2(t)$.

The ecological model for the minimal cancer switch with two phenotypes writes

$$\frac{dC_1}{dt} = r_1 C_1 - w_{12} C_1 + w_{21} C_2 \tag{1}$$

$$\frac{dC_2}{dt} = r_2 C_2 - w_{21} C_2 + w_{12} C_1 \tag{2}$$

and yields an exponential growth solution:

$$C_1(t) = e^{\gamma t}\left(\alpha_1 e^{\varphi t} + \beta_1 e^{-\varphi t}\right), \qquad C_2(t) = e^{\gamma t}\left(\alpha_2 e^{\varphi t} + \beta_2 e^{-\varphi t}\right) \tag{3}$$

with

$$\gamma = \frac{1}{2}\Big(r_1 + r_2 - (w_{12} + w_{21})\Big), \qquad \varphi = \frac{1}{2}\sqrt{[(r_1 - r_2) - (w_{12} - w_{21})]^2 + 4 w_{12} w_{21}} \tag{4}$$

and

$$\alpha_1 = \frac{c_1(0)(\gamma + \varphi + w_{21} - r_2) + c_2(0) w_{21}}{2\varphi}, \qquad \beta_1 = \frac{-c_1(0)(\gamma - \varphi + w_{21} - r_2) - c_2(0) w_{21}}{2\varphi} \tag{5}$$

$$\alpha_2 = \frac{c_2(0)(\gamma + \varphi + w_{12} - r_1) + c_1(0) w_{12}}{2\varphi}, \qquad \beta_2 = \frac{-c_2(0)(\gamma - \varphi + w_{12} - r_1) - c_1(0) w_{12}}{2\varphi} \tag{6}$$

Despite exponential growth, for fixed parameters, the system goes towards a stable phenotypic compositions. For the two dimensional model, analytical solutions can be computed [1,2]. Since $exp(-\varphi t) \to 0$, we can compute:

$$\lim_{t\to\infty} \frac{C_2(t)}{C_1(t)} = \frac{\alpha_2}{\alpha_1} = \frac{\gamma + \varphi - (r_1 - w_{12})}{w_{21}} \tag{7}$$

As seen in figure S1, this result is equivalent to a Moran process model, where a CPC is taken into account: the stable distribution is easy to compute if we define a system for the proportion of phenotypes. Let us redefine, without loss of generality, c_i to be the fraction of phenotype i in the population, $C_i/\sum_j C_j$, and so $\sum_j c_j = 1$. The model now reads

$$\frac{dc_1}{dt} = c_1 r_1 - c_1 w_{12} + w_{21} c_2 - c_1 \phi(c_1, c_2) \tag{8}$$

$$\frac{dc_2}{dt} = c_2 r_2 - c_2 w_{21} + w_{12} c_1 - c_2 \phi(c_1, c_2) \tag{9}$$

---

* corresponding author

The competition term $\phi(c_1, c_2) = r_1 c_1 + r_2 c_2$ is the average growth rate of the population and ensures $c_1 + c_2 = 1$. The system has an attractor state that is independent of initial conditions

$$\begin{aligned} c_1^* = \frac{C_1^*}{C_1^* + C_2^*} =& \frac{(r_1 - r_2) - (w_1 + w_2)}{2(r_1 - r_2)} \pm \\ & \pm \frac{\sqrt{[(r_1 - r_2) - (w_1 + w_2)]^2 + 4(r_1 - r_2) w_{21}}}{2(r_1 - r_2)} \\ c_2^* = \frac{C_2^*}{C_1^* + C_2^*} =& 1 - c_1^*. \end{aligned} \tag{10}$$

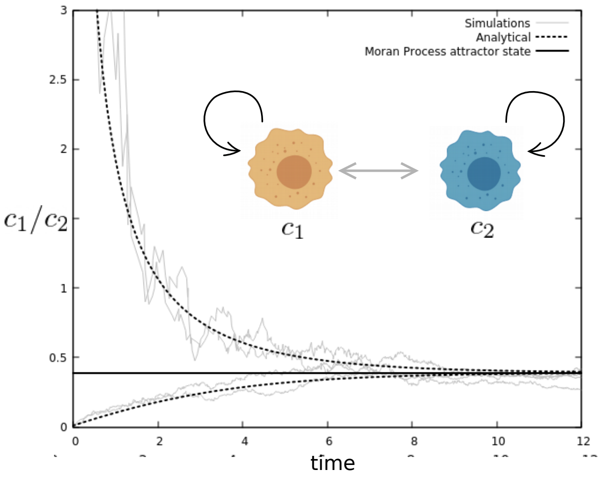

Fig. S1. Any initial configuration recapitulates the original tumor composition. The c_1/c_2 cell type initial distributions evolve into the same equilibrium configuration. Gillespie simulations (grey), model (3) solution (dashed) and Moran process (6) attractor state (filled line) predict phenotypic evolution. These results are consistent with phenotypic stability being an experimental marker for the existence of stochastic switching [3,4].

B. Phenotypic switching architectures are resistant to cytotoxic therapy

In this section we describe the mathematical background of the replication threshold of equation (11) in the main text. This scenario is understood by computing the Jacobian matrix for the $(0, 0)$ attractor

$$J^{(0,0)}_{C_1,C_2} = \begin{pmatrix} r_1 - w_{12} & w_{21} \\ w_{12} & r_2 - w_{21} \end{pmatrix} \tag{11}$$

We know that stability follows from the eigenvalues of $J^{(0,0)}_{C_1,C_2}$ being negative, which results in two conditions

$$\text{trace}(J) = (r_1 + r_2) - (w_{12} + w_{21}) < 0? \tag{12}$$

$$\det(J) = r_1 r_2 - r_1 w_{21} - r_2 w_{12} > 0? \tag{13}$$

from where the r_1, r_2 plane can be drawn to understand how a replicating phenotype can sustain a secondary one even if the latter is targeted by therapy (Fig. S2).

C. Sequential therapy: Targeting multiple phenotypes ($N > 2$)

As seen for glioblastoma [4] or breast cancer [5], tumors usually take advantage of stochastic switching between more than two phenotypes. Analytical treatment becomes costly, but simple gillespie simulations can still be performed to

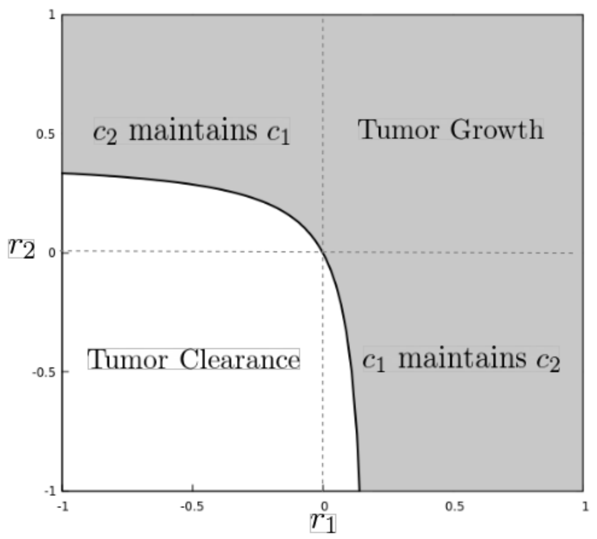

Fig. S2. Stability conditions for the cancer-free scenario highlight how a replicating phenotype can still drive tumor growth even if the other phenotype is targeted by therapy so that its *effective* growth rate is negative.

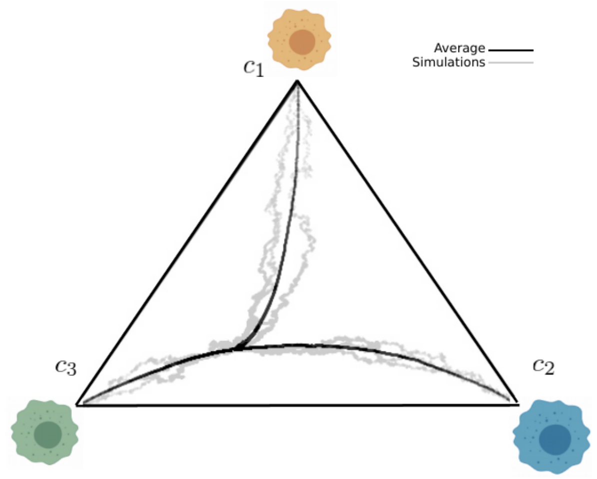

Fig. S3. Evolution of a 3-phenotypes tumor towards a stable phenotypic composition. Each vertex of the triangle corresponds to the tumor harboring 100% of the cells of a given phenotype. As for experimental evidence of phenotypic switching in tumors, any starting cell recapitulates the same stable composition, independent of initial conditions and only dependent on system parameters r_i, w_{ij}.

see how phenotypic composition in our model evolves to a stable state independently of initial conditions (Fig. S2), as seen in experimental approaches.

Despite each phenotype can have different replication and transition rates, and each therapy has different effects, we can get a glimpse of the average effect of sequential therapy by making an coarse-graining assumption: all replicating and dying cells do so at equal rates, r_+ and r_- respectively, and transition rates between replicating and dying cells are also homogeneous.

In this scenario, suppose a system with 2 phenotypes that replicate at $r_+ > 0$ and hold a dying phenotype $r_- < 0$:

$$\begin{cases} \dot{C}_1 = & C_1 r_+ - C_1 w_{++} + w_{++} C_2 - C_1 w_{+-} + C_3 w_{-+} \\ \dot{C}_2 = & C_2 r_+ - C_2 w_{++} + w_{++} C_1 - C_2 w_{+-} + C_3 w_{-+} \\ \dot{C}_3 = & C_3 r_- - C_3 w_{-+} + C_2 w_{+-} - C_3 w_{-+} + C_1 w_{+-} \end{cases} \tag{14}$$

Let us now call σ_+ the sum of positive populations, $\sigma_+ = C_1 + C_2$, the system reduces to

$$\begin{cases} \dot{\sigma_+} = & \sigma_+ r_+ - \sigma_+ w_{+-} + 2C_3 w_{-+} \\ \dot{C_3} = & C_3 r_- - 2C_3 w_{-+} + \sigma_+ w_{+-} \end{cases} \tag{15}$$

which following section A from the appendix results in a minimal threshold for the positive population replication rate

$$r_+^* = \frac{w_{+-}}{1 + 2\dfrac{w_{-+}}{|r_-|}}. \tag{16}$$

The same minimal scenario can be studied for the case where a single r_+ replicator holds two negative replicators ($i.e$ replicators with negative effective growth rate, $r_- = b_- - d_- < 0$). The mean field model for this metapopulation reads

$$\begin{cases} \dot{C_1} = & C_1 r_+ - C_1 w_{+-} + w_{-+} C_2 - C_1 w_{+-} + C_3 w_{-+} \\ \dot{C_2} = & C_2 r_- - C_2 w_{-+} + w_{+-} C_1 - C_2 w_{--} + C_3 w_{--} \\ \dot{C_3} = & C_3 r_- - C_3 w_{--} + C_2 w_{--} - C_3 w_{-+} + C_1 w_{+-} \end{cases} \tag{17}$$

Once again, we can compact the negative populations into $\sigma_- = C_2 + C_3$. The model can be expressed as

$$\begin{cases} \dot{C_1} = (r_+ - 2w_{+-})c_1 + w_{-+}\sigma_- \\ \dot{\sigma_-} = \sigma_- r_- - \sigma_- w_{-+} + 2c_1 w_{+-} \end{cases} \tag{18}$$

There is again a threshold value for the needed replication rate of c_1 to maintain the dying compartment, σ_-:

$$r_+^* = 2\frac{w_{+-}}{1 + \frac{w_{-+}}{|r_-|}}. \tag{19}$$

Interestingly, equations (12) and (15) indicate that the effects of increasing the number of dying or replicating phenotypes in the switching architecture are different. The same mathematical procedure can be repeated for n_+ replicating populations and n_- dying populations, collapsing them into the sum of σ_+ and σ_- and obtaining a function for how the cost of maintainance depends on the number of phenotypes that we can target by therapy (Fig. 4 of the main text)

$$r_+^*(n_+, n_-) = n_- \frac{w_{+-}}{1 + n_+ \frac{w_{-+}}{|r_-|}}. \tag{20}$$

D. The (n, c_1, c_2) system: a PHS tumor competing in a healthy tissue environment

We have not yet considered the conditions for healthy tissue colonization. What is the cost of maintaining a stochastic strategy when chromatin-stable cells populate the system? A minimal step is considering competition against the non-switching original tissue. We can consider the normal tissue a simple logistically self-renewing population n. Our system now reads

$$\begin{aligned} \frac{dn}{dt} =& r_n n - n\phi(n, c_1, c_2) \\ \frac{dC_1}{dt} =& c_1 r_1 - c_1 w_{12} + w_{21} c_2 - c_1 \phi(n, c_1, c_2) \\ \frac{dC_2}{dt} =& c_2 r_2 - c_2 w_{21} + w_{12} c_1 - c_2 \phi(n, c_1, c_2) \end{aligned} \tag{21}$$

We face now a new set of attractors for the dynamics of population fractions with $N = n + c_1 + c_2 = 1$. The simplest non-trivial attractor $\varphi_n = (n = 1, 0, 0)$ defines the homeostatic tissue state. Under which conditions can the cancer population invade it?

The Jacobian of the system is crucial for understanding the stability of the different attractor states

$$J_{n,c_1,c_2} = \begin{pmatrix} r_n(1-2n) - r_1c_1 - r_2c_2 & -nr_1 & -nr_2 \\ -c_1r_n & r_1 - w_{12} - r_nn - r_2c_2 - 2r_1c_1 & w_{21} - c_1r_2 \\ -c_2r_n & w_{12} - c_2r_1 & r_2 - w_{21} - r_nn - r_1c_1 - 2r_2c_2 \end{pmatrix} \tag{22}$$

The conditions for the $(0,0,0)$ stability are obvious, $r < 0$ together with the original phenotypic switching conditions. The Jacobian for the healthy-tissue stability is

$$J_{(n=1),0,0} = \begin{pmatrix} -r_n - \lambda & -r_1 & -r_2 \\ 0 & r_1 - w_{12} - r_n - \lambda & w_{21} \\ 0 & w_{12} & r_2 - w_{21} - r_n - \lambda \end{pmatrix} \tag{23}$$

We find the eigenvalues from

$$\det(J_{(1,0,0)}) = -(r+\lambda) \begin{vmatrix} r_1 - w_{12} - r_n - \lambda & w_{21} \\ w_{12} & r_2 - w_{21} - r_n - \lambda \end{vmatrix} = 0 \tag{24}$$

The first is obvious, $r_n > 0$. The second condition is more strict, $r_n > (r_1 + r_2 - (w_{12} + w_{21})/2$, meaning that the tissue must replicate at a rate higher than the average *effective* rate of the switching attractor.

To obtain a better understanding of the stability space of $(1,0,0)$, we can consider a limit case scenario with $w_{ij} = w$, and

$$J^{w_{ij}=w}_{(n=1,0,0)} = \begin{pmatrix} -r_n - \lambda & -r_1 & -r_2 \\ 0 & r_1 - w - r_n - \lambda & w \\ 0 & w & r_2 - w - r_n - \lambda \end{pmatrix} \tag{25}$$

The first eigenvalue is again $\lambda_1 = -r_n$. The other two simplify to

$$\lambda_{i,j} = \frac{1}{2}\left(-b \pm \sqrt{b^2 - 4c}\right) \tag{26}$$

with

$$b = 2r + 2w - r_1 - r_2 \tag{27}$$

$$c = r_1r_2 + r^2 + rw - r(r_1 + r_2) - w(r_1 + r_2) \tag{28}$$

In this context, if $b > 0$, we can have either stability (for $c > 0$) or instability in one of the two $\vec{c_1}, \vec{c_2}$ directions (for $c < 0$). If $b < 0$, either one direction is unstable ($c < 0$) or both ($c > 0$), meaning that both cancer populations will grow together. The $c < 0$ scenarios relate with those parameter combinations in which one population will grow, holding the other despite this second one cannot compete against the healthy replicator (Fig. S3). They can be written as a condition for the minimal value that the self-renewal capacity of the tissue has to reach to evade the plastic attractor, provided that $r_n > 0$ and $r_1 + r_2 > w_{12} - w_{21}$:

$$\begin{aligned} r_n > &\frac{1}{2}(r_1 + r_2 - w_{12} - w_{21}) + \\ &+ \frac{1}{2}\sqrt{\left((r_1 - r_2) - (w_{12} - w_{21})\right)^2 + 4w_{12}w_{21}} \end{aligned} \tag{29}$$

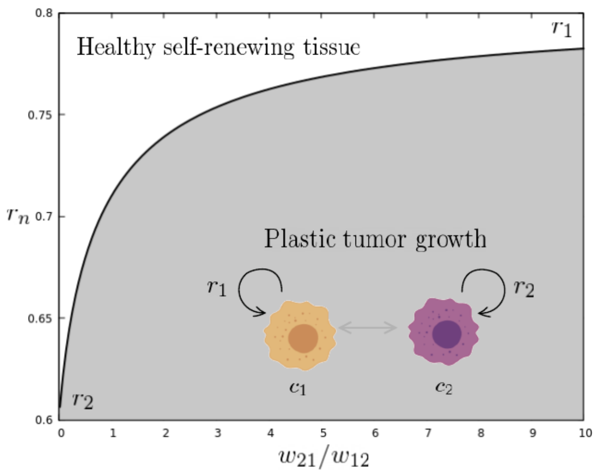

Fig. S4. Minimal healty tissue self-renewal r_n to avoid the growth of a malignant switching population. In this particular case, $r_1 > r_2$. As w_{21}/w_{12} increases, the c_1/c_2 proportion increases as well, and the healthy tissue needs a higher replication rate as the presence of c_1 increases. This is significative, as it points out that a good replicator, as for an oncogene-mutated phenotype, could hold a worse-adapted one through switching. The relevance of the w_{21}/w_{12} tuning is indicative of potential therapies not only targeting oncogenic pathways but also transdifferentation rates.

E. Gillespie Simulations

Simulations to compare with analytical results (Figures 4 and 5 in the main text) are built in `python`. In it, A Gillespie algorithm is used to generate the stochastic birth-switch-death process, where the probability of each event is related with its rate through $P_j \sim r_j/\sum_i r_i$. With this, we obtain time trajectories for the amount of each population and the total cancer population.

In figure 4, we want to assess the value for r_+ able to maintain the cancer population. Due to stochasticity and small initial conditions, some values of $r < r_+$ are able to escape population extinction, and some values of $r > r_+$ end up in zero population. To take this into account, we have made 100 runs for each scenario, and taken the value of r_+ as the replication rate for which 95% of the runs ended up in population extinction. The error bars in figure 4 (main text) contemplate the values for which 100% of the runs end up in extinction (lower bound) or only 90% of the runs end up in extinction (upper bound).

In figure 5, the time trajectory of population size for each phenotype allows us to compute the phenotypic composition trajectory $c_1(t)/c_2(t)$. This is computed for different initial population distributions to show how the system always evolves to the same stable distribution given a fixed set of parameters r_i, w_{ij}.

Bibliography

1. Balaban NQ, Merrin J, Chait R, Kowalik L, Leibler S. 2004 Bacterial persistence as a phenotypic switch. Science 305, 1622–1625
2. Gunnarsson E, De S, Leder K, Foo J. 2020 Understanding the role of phenotypic switching in cancer drug resistance. Journal of Theoretical Biology, 110162.
3. Neftel C, *et al.* 2019 An integrative model of cellular states, plasticity, and genetics for glioblastoma. Cell 178, 835–849.
4. Gupta PB, Fillmore CM, Jiang G, Shapira SD, Tao K, Kuperwasser C, Lander ES. 2011 Stochastic state transitions give rise to phenotypic equilibrium in populations of cancer cells. Cell 146, 633–644.
